# The Effects of Online Home-Based Pilates Combined with Diet on Body Composition in Women Affected by Obesity: A Preliminary Study

**DOI:** 10.3390/nu16060902

**Published:** 2024-03-21

**Authors:** Francesca Greco, Maria Grazia Tarsitano, Loretta Francesca Cosco, Federico Quinzi, Katia Folino, Marco Spadafora, Moomna Afzal, Cristina Segura-Garcia, Samantha Maurotti, Roberta Pujia, Arturo Pujia, Pasqualina Buono, Gian Pietro Emerenziani

**Affiliations:** 1 Department of Movement, Human and Health Sciences, Foro Italico University of Rome, 00135 Rome, Italy; f.greco2@studenti.uniroma4.it; 2 Department of Medical and Surgical Science, University Magna Grecia, 88100 Catanzaro, Italy; mariagrazia.tarsitano@unicz.it (M.G.T.); segura@unicz.it (C.S.-G.); roberta.puj@gmail.com (R.P.); pujia@unicz.it (A.P.); 3 Department of Medicine, Movement Sciences and Wellbeing, University Parthenope, 80133 Naples, Italy; lorettafrancesca.cosco001@studenti.uniparthenope.it (L.F.C.); buono@uniparthenope.it (P.B.); 4 Department of Clinical and Experimental Medicine, University Magna Grecia, 88100 Catanzaro, Italy; katia.folino@unicz.it (K.F.); marco.spadafora@unicz.it (M.S.); moomna.afzal@studenti.unicz.it (M.A.); smaurotti@unicz.it (S.M.); emerenziani@unicz.it (G.P.E.); 5 Research Center for the Prevention and Treatment of Metabolic Diseases, University Magna Grecia, 88100 Catanzaro, Italy; 6 CEINGE-Biotecnologie Avanzate Franco Salvatore s.r.l, 80131 Naples, Italy

**Keywords:** online training, fat mass, exercise, diet, health

## Abstract

Diet and exercise intervention are the first strategies to counteract obesity. An online home-based exercise program may be a feasible approach in an obese population. Therefore, this study aimed to investigate the effects of twelve weeks of online Pilates plus diet on body composition in individuals affected by obesity. Thirty-five females were randomly assigned to a home-based Pilates group (PG, *n* = 18) or a group without intervention (control group, CG, *n* = 17). All participants followed a Mediterranean diet. The PG followed a twelve-week online Pilates Matwork program (three times/week; 180 min/week), while the CG was not involved in any structured physical exercise program. Body composition and handgrip strength were evaluated at baseline (T_0_) and after the intervention (T_1_). A significant group × time interaction (*p* < 0.05) was found for the fat mass percentage (pFM). Specifically, the pFM was significantly lower at T_1_ than at T_0_ in the PG. Significant group × time interactions for fat-free mass (*p* < 0.05), appendicular skeletal muscle mass (*p* < 0.05), and skeletal muscle mass (*p* < 0.01) were found. All these variables were significantly higher at T_1_ than at T_0_ in the PG (*p* < 0.05). Home-based Pilates combined with diet intervention may represent an effective strategy to improve body composition in terms of fat mass reduction and muscle mass gain in adults affected by obesity.

## 1. Introduction

Overweight and obesity, defined as an abnormal or excessive accumulation of body fat, are major risk factors for the onset of different diseases (e.g., cardiovascular diseases, diabetes, and several types of cancer) [1]. Obesity is a multifactorial disease which presents different phenotype peculiarities [2,3], and it is a worldwide health challenge that negatively influences the quality of life of people and burdens national healthcare systems financially [1]. The main cause of obesity is due to an increase in caloric intake and a decrease in physical activity (PA) levels. Specifically, a hypocaloric diet can lead to the recommended weight loss of ≥5% of an individual’s total body weight [4]. However, caloric restriction not only reduces regional and total fat mass but may also lead to a significant decrease in lean body mass [5]. This can result in a reduced metabolic rate which complicates further weight loss or weight maintenance [6].

Moderate-intensity PA without dietary intervention generally leads to modest weight loss and seems sufficient to prevent weight gain in adults [7]. However, PA combined with moderate dietary restrictions provides greater weight loss than diet alone [7]. Previous studies showed that aerobic training plus diet may positively affect body composition and physical fitness components in individuals affected by obesity [8,9]. Specifically, a significant decrease in body mass and the percent of fat mass as well as improvements in physical performance were revealed [8,9]. It is recommended that overweight and obese individuals participate in at least 150 min of PA per week to elicit modest reductions in body weight [8]. This emphasizes that the combination of exercise and diet may lead to the best results regarding body composition and physical fitness improvement as regular PA combined with dietary interventions is recommended as one of the most effective prevention and treatment options [1,10].

Although the health-related benefits of regular exercise are well known, physical inactivity levels are still high and tend to increase with age [11]. Physical inactivity prevalence for on-site training protocols increases, especially in overweight and obese individuals, due to several difficulties [12] (e.g., physical discomfort, difficulties in reaching sports facilities, and a lack of time). Therefore, alternative training methods (e.g., online training) should be sought for the treatment of obesity and to facilitate PA promotion in this population. In addition, after the COVID-19 pandemic, the use of online training sessions may represent a new strategy to stimulate behavioural changes. Online training consists of a mix of supervised and unsupervised training modalities (i.e., pre-registered lessons) and represents a valid strategy to overcome barriers to PA among this population [12,13]. Perri and colleagues [14] reported that individuals affected by obesity who exercise at home have higher adherence to exercise and/or lose more body mass compared with on-site training (e.g., health clubs). In this context, since digital technologies play a key role in everyday life, they may represent a low-cost and time-saving strategy to facilitate PA promotion. Therefore, online training protocols may be a feasible alternative to the traditional exercise prescription in overweight and obese individuals. Pilates is an exercise method widely used for improving health-related outcomes in several clinical conditions [15,16]. Pilates is a low-cost body/mind training method that requires balance, strength, flexibility, and muscle control [17]. Most importantly, it has a low impact on joints since the exercises can be performed in sitting, standing, or lying positions. All these characteristics make Pilates very attractive to individuals affected by obesity [18]. Moreover, the positive psychophysiological effects of Pilates in overweight and obese individuals have been highlighted [19].

Although not designed to reduce body mass, Pilates may represent a valid option for overweight or obese individuals who have difficulties with traditional physical exercise programs [20]. Previous studies reported positive effects of supervised on-site Pilates on body mass, body mass index, and fat mass in individuals affected by obesity [19,21,22]. To the best of our knowledge, only one recent study reported significant improvements in several physical fitness components after 12 weeks of online Pilates [13]. However, that study considered only a Pilates intervention without diet restriction [13]. The Mediterranean diet is a well-known nutritional intervention therapy used to manage obesity, and a supervised home-based Pilates program that could be always accessible to participants may represent an effective approach to promoting participation in physical activity in this population. Therefore, this study aimed to investigate the effects of online home-based Pilates training and dietary intervention on body mass and body composition in adults affected by overweight or obesity.

## 2. Materials and Methods

### 2.1. Study Design

This study adopted a randomized control trial design. Participants were enrolled at the Clinical Nutrition and the Clinical Research Treatment of Eating Disorders Units of the “Renato Dulbecco” University Hospital, Catanzaro, Italy. All participants were physically evaluated at the Physical Exercise and Sports Science laboratory of “Magna Graecia” University, Catanzaro, Italy.

**Figure 1:**
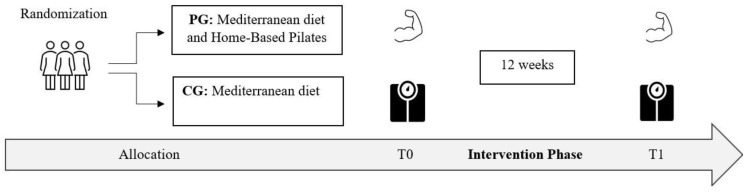
Study design flowchart. PG, Pilates group; CG, control group; T_0_, baseline; T_1_, after twelve weeks of intervention.

After the baseline assessment (T_0_), participants were randomly assigned to a home-based Pilates group (PG) or a group without intervention (control group, CG). The PG followed a twelve-week online Pilates Matwork program (three times/week; 180 min/week), while the CG members were not involved in any structured physical exercise program and maintained their usual lifestyle routines. Both groups followed the same Mediterranean diet protocol. Body composition, anthropometric characteristics, and grip strength were evaluated pre (T_0_) and post (T_1_) intervention in the morning and at the same time during the day. The study design adopted is reported in Figure 1.

All experimental procedures detailed in the following paragraphs comply with the Declaration of Helsinki. After being informed of the research procedures, goals, and risks and the benefits of participating in this study, the participants signed a written informed consent form approved by the ethical committee of Regione Calabria (approval number n. 396/19 November 2020).

### 2.2. Participants

An a priori sample size estimation performed using G-Power 3.1.9.2 showed that twenty participants were sufficient to achieve a statistical power of 1 − β = 0.80 (an effect size of 0.35; α = 0.05). To account for the possible drop-out rate, as reported in a recent study focusing on online training [14], thirty-five female individuals affected by overweight/obesity were recruited according to eligibility criteria. The participants were randomly assigned to the PG (*n* = 18) or CG (*n* = 17).

As fifteen participants withdrew from the study (PG, *n* = 7; CG, *n* = 8) during the twelve weeks, twenty participants (mean ± SD; age: 47.7 ± 10.7 years; BMI: 33.1 ± 4.7 kg/m^2^; physical activity level: 2184.2 ± 4367.8 MET/week, using the Global Physical Activity Questionnaire [23]) completed the intervention protocol (PG, *n* = 11; CG, *n* = 9). All participants were Italian Caucasian women with no previous history of structured PA or dietary interventions in the 6 months preceding the study. During the medical screening, three participants in the PG and three in the CG reported being in menopause. Moreover, most of our participants had an intermediate educational level. The baseline characteristics of the participants that completed the intervention protocol (*n* = 20) are reported in Table 1.

Inclusion criteria were females aged between 25 and 64 years old with a BMI ≥ 25 kg/m^2^, while exclusion criteria were the current use of anti-obesity drugs, pregnancy, types 1 and 2 diabetes mellitus, latent autoimmune diabetes, chronic renal failure, active or severe infections, liver failure, recent major cardiovascular events, unstable angina, heart failure (NYHA III–IV) and respiratory failure, cardiac arrhythmias, and neoplastic diseases.

### 2.3. Diet and Exercise Program

The Mediterranean diet was based on the total daily energy expenditure of each participant, with nutrients subdivided according to the Mediterranean Diet scheme using five meals (breakfast, morning and afternoon snacks, and dinner). The traditional diet focuses on vegetables, fruits, whole cereals, and legumes, increasing fibre consumption. Lean proteins are acquired from fish and poultry, and extra virgin olive oil is the principal source of healthy fat. The Mediterranean diet includes a high-carbohydrate assumption (50–60% of daily energy requirements) and a low-fat regimen (no more than 30% of total energy), emphasizing caloric restriction. The macronutrient dietary composition consisted of a daily intake of 30% of calories coming from fat, 20–25% from protein, and 45–50% from carbohydrates.

**Figure 2:**
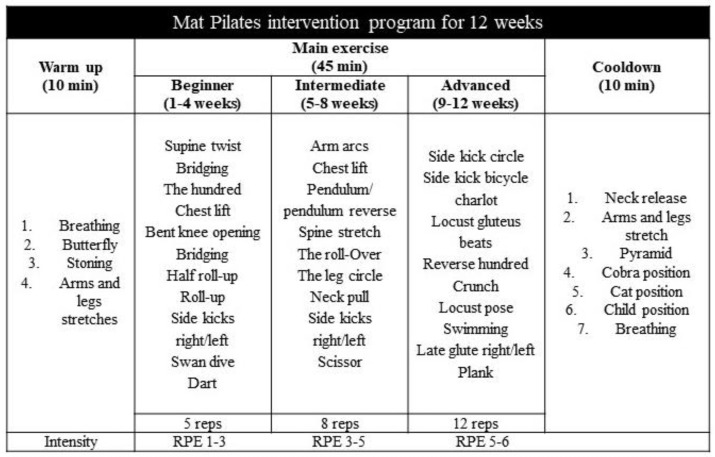
The mat Pilates program adopted in this study. RPE, rate of perceived exertion; reps, repetitions.

The online home-based Pilates Matwork training program was performed via a virtual class using Microsoft^®^ Teams (version: 19.1.8). Specifically, the PG participants performed three Pilates classes a week (one supervised and two unsupervised), each lasting one hour, for a total of 12 weeks. Each class was composed of warm-up exercises (10 min. of breathing and total body activation); Pilates Matwork (45 min. of general conditioning with the traditional Pilates repertoire of exercises for beginners and intermediates), and a cooldown (5 min. of stretching and respiratory exercises). Some of the traditional exercises adopted for the central phase included the hundred, roll up, roll over, scissors, bridge, side kick, single-leg circle, and swimming. When necessary, simpler exercise variants were adopted in case a participant was initially unable to perform the movement correctly or did not feel comfortable. The mat Pilates program adopted across the 12 weeks of intervention is reported in Figure 2.

All Pilates exercises were performed in a single series of repetitions, and the number of repetitions was increased according to the participant’s ability to complete the exercise (from 5 to 12 repetitions across the 12 weeks). Moreover, the use of a Pilates soft ball was introduced after 8 weeks. During the supervised Pilates classes, practitioners were followed by a certified Pilates instructor. Each supervised online class was recorded and regularly updated every week. Specifically, the supervised classes were accessible to the participants to allow them to replicate the exercises during the other two unsupervised classes. The intensity of Pilates was evaluated using the Rate of Perceived Exertion scale (Borg CR-10) (RPE) [24] at the end of each class. The scale scores perceived effort from 0 (no effort at all) to 10 (the absolute maximum) [24]. Participants were instructed in the use of the online platform during the baseline assessment, and adherence to exercise and diet or diet alone was assessed weekly for each group (PG and CG) using an Excel sheet form. For the PG, exercise and diet were monitored at each supervised class by the Pilates instructor, whereas for the CG, a medical doctor monitored adherence to the diet program through a phone call, and participants were reminded to not change their lifestyle routines.

### 2.4. Physical Fitness Assessments

At baseline (T_0_) and after 12 weeks of the intervention (T_1_), the participants underwent a physical fitness examination which involved the following measurements:

- Height was measured barefoot to the nearest 0.1 cm using a stadiometer (SECA, Intermed S.r.l., Milano, Italy). Participants stood with their body mass evenly distributed on both feet, heels together, and head positioned in a midline position (Frankfort horizontal plane), with their arms hanging loosely. To obtain a reliable measurement, participants were asked to inhale deeply and stretch to their full height. Three measurements of height were carried out.
- Body mass and body composition were measured using a bioelectrical impedance method (BIA ACCUNIQ 360, Daejeon, Republic of Korea) while participants wore minimal clothing and removed any metal accessories before the analysis was conducted. After entering the participant’s height, date of birth, and gender into the device, their body mass was automatically measured. During the measurement, participants stood in an upright position, with arms at about a 30° angle away from the trunk. This position was maintained until the end of the measurement, and no talking or movements were allowed. The variables of interest were the body mass index (BMI = body mass (kg)/height^2^ (m)^2^), the fat mass (pFM) expressed as a percentage of body mass, the fat-free mass (FFM) expressed in kilograms, and the appendicular skeletal muscle mass (ASMM) and skeletal muscle mass (SMM) expressed in kilograms. Body composition assessments were always conducted in the morning and at the same time during the day. Participants were asked to refrain from eating or drinking 3 to 4 h before the assessment.
- A handgrip strength test (HGS) was used to evaluate the maximum isometric strength of the hand and forearm muscles utilizing a handgrip dynamometer (Jamar Hydraulic Hand Dynamometer, Sammons Preston Rolyan, Bolingbrook, IL, USA) [25]. Participants were instructed to squeeze the dynamometer with their hand as hard as possible for three seconds, maintaining the elbow at a right angle, while seated on a standard armless chair. The width of the handle was adjusted for each participant according to their hand size (i.e., the middle phalanx on the inner handle). Three trials interspersed with 30 s of rest were performed for each hand, and the mean of the right and left hand results was used for further analysis.

### 2.5. Statistical Analysis

A statistical analysis was conducted using IBM^®^ SPSS Statistics software version 23.0 (SPSS Inc., Chicago, IL, USA). The normal distribution of the dependent variables (body mass, BMI, pFM, FFM, ASMM, SMM, and HGS) was tested using the Shapiro–Wilk test. As all variables were normally distributed, a parametric analysis was conducted. The similar baseline characteristics of the two groups were verified using an unpaired *t*-test at T_0_. For each variable, a two-way (group × time) ANOVA for repeated measures on time was used to detect the significant effects of two main factors: group (PG vs. CG) and time (T_0_ vs. T_1_). A Bonferroni post hoc analysis was carried out when significant differences were detected. The level of significance was set at *p* < 0.05.

## 3. Results

**Figure 3:**
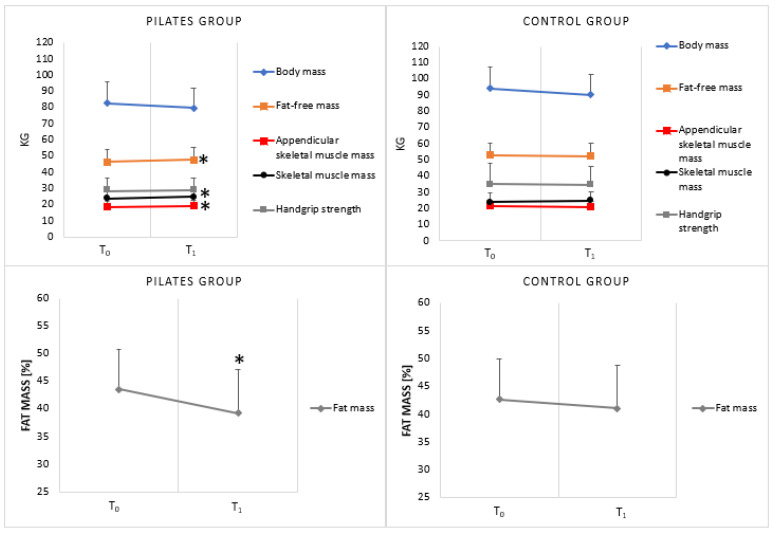
Results of outcome measures. T_0_, baseline; T_1_, after 12 weeks of intervention. * Denotes significant differences between T_0_ and T_1_.

Twenty participants completed the intervention protocol, reporting no adverse effects. Percentages of adherence to the PG and CG protocols were 64% and 78%, respectively. In the PG group, RPE values ranged between 4 and 5. Results for body mass, pFM, FFM, ASMM, SMM, and HGS in both groups (PG and CG) are graphically reported in Figure 3.

The mixed-model ANOVA showed a significant group x time interaction on the pFM *(F*_1,18_ = 5.824, *p*< 0.05, *η*^2^ *=* 0.244*)*, FFM *(F*_1,18_ *=* 7.203, *p* < 0.05, *η*^2^ *=* 0.286*)*, ASMM (*F*_1,18_ *=* 6.384, *p* < 0.05, *η*^2^ *=* 0.262), and SMM (*F*_1,18_ *=* 8.938, *p* < 0.01, *η*^2^ *=* 0.332). A post hoc analysis showed that the pFM was significantly lower at T_1_ than at T_0_ in the PG (*p* < 0.05), while no significant difference was observed in the CG (Table 2). Moreover, the FFM, ASMM, and SMM were significantly higher at T_1_ than at T_0_ in the PG (*p* < 0.05) while no significant difference was found in the CG (Table 2). A significant main effect of time on body mass (*F*_1,18_ = 29.960, *p* < 0.01, *η*^2^ *=* 0.625), BMI (*F*_1,18_ = 34.941, *p* < 0.01, *η*^2^ *=* 0.660) and the pFM (*F*_1,18_ *=* 30.048, *p* < 0.01, *η*^2^ *=* 0.625) was found. The post hoc analysis showed that body mass, BMI, and the pFM were significantly lower at T_1_ than at T_0_ (*p* < 0.01) (Table 2). Moreover, no significant difference was found for the HGS variable (Table 2). No significant main effect of group was observed for any of the variables of interest.

## 4. Discussion

This study aimed to investigate the effects of online home-based Pilates training on body mass and body composition in a group of female adults affected by obesity and involved in a Mediterranean diet program. Our results showed that only participants involved in the online home-based Pilates classes combined with diet showed an increase in fat-free mass and appendicular and skeletal muscle mass and a reduction in the percentage of fat mass after training compared to the pre-intervention assessment. Therefore, Pilates may represent an effective exercise strategy combined with a traditional nutritional protocol to improve body composition in women affected by obesity. Notwithstanding the increase in the ASMM, and SMM observed in the PG, neither intervention induced modifications in grip strength.

Although the exercise administration was supervised weekly, the drop-out rate (43%) confirms that the modification of lifestyle habits and especially the incorporation of training into everyday life remains challenging in a population affected by obesity. This high drop-out rate advocates for the adoption of adjuvant strategies to increase exercise adherence in this population, like social cognitive theory [26]. However, our drop-out rate was lower than that reported in a previous investigation (~60%) when individuals affected by obesity performed an individualized exercise program [8]. Therefore, online training may facilitate PA promotion in this population.

Previous investigations reported positive effects of on-site Pilates on the BMI and fat mass of individuals affected by obesity [19,21,22]. Moreover, a recent systematic review reported that 8–24 weeks of on-site Pilates practice reduces body mass, BMI, and body fat percentage in adults with overweight or obesity [20]. Our results are partially in agreement with these studies. Indeed, while we did not observe a reduction in body mass, we observed a reduction in body fat percentage, which represents an important indicator for reducing health-related risk factors [27]. The unaltered body mass and BMI could be explained by the modifications in body composition observed following training and in particular by the increases in the FFM, ASMM, and SMM. Body composition is one of the fitness components related to health which facilitates a better understanding of obesity-related complications [28]. However, these effects were not observed with the nutritional intervention only. Therefore, diet alone was not effective in inducing positive effects on body composition. Consequently, in the present study, we showed that combining dietary intervention with Pilates is a valid strategy to positively influence body composition in individuals affected by obesity. Our results are in contrast with previous studies which revealed no positive effects on lean mass after on-site Pilates interventions [19,29] as well as with a recent study reporting no improvements after 12 weeks of online Pilates [13]. These differences may be accounted for by the adoption of an exercise intervention without a dietary intervention. Indeed, Park et al. [13] did not include a dietary intervention as a control group or a combination of exercise and diet. It is well known that a hypocaloric diet alone is effective in the reduction of body mass, BMI, and the percentage of fat mass [30]. However, only in the PG did we observe a significant fat mass reduction, highlighting the important role of exercise plus dietary interventions as a primary strategy to counteract obesity [10].

In both groups, no differences in grip strength as an indicator of overall health [31] were observed after twelve weeks. It has been suggested that free weights and resistance exercise machines are the two major methods of increasing strength [32]. Moreover, to increase muscle strength, exercise intensity should be higher than 60% of one maximal repetition [32]. In light of these considerations, it may be hypothesized that two factors account for the lack of improvement in HGS observed in our study: (1) the type of exercise proposed mainly involved lower limbs and core muscles; (2) the low intensity of the strength exercise proposed.

In the CG, body composition variables showed a slight improvement despite the fact that no significant differences were observed. This could be explained by the low rate of compliance with the diet program. Indeed, although we monitored participants’ adherence to the diet program through a weekly phone call, we could not establish whether the participants’ answers were accurate.

Overall, Pilates could be considered an alternative exercise modality in individuals affected by obesity who have difficulties engaging in traditional exercise protocols, although no superior effects were observed in terms of body composition when compared with other traditional types of exercise [33]. However, further controlled trials are needed to elucidate the effectiveness of Pilates for treating overweight and obesity [20]. Further investigations on the effects of the combination of Pilates and different dietary regimens, like protein supplementation, on body composition are needed as they may facilitate better guidance on metabolic complications associated with obesity [28].

We are aware of some study limitations. We did not use strategies based on social cognitive theory, such as a constant incentive, phone and email reminders, or personal booster sessions to encourage and support adherence. However, this choice helped us to increase the ecological validity of the study, rendering it more representative of adherence to an online Pilates course. Moreover, we did not assess metabolic parameters or hormonal profiles to deeply analyse the effects of the Pilates and diet interventions. Finally, further studies with a bigger sample size, a narrow age range, and different menopause stages should compare the effects of online and on-site training formats with comparable exercise intensities on body composition in individuals affected by obesity.

## 5. Conclusions

Home-based Pilates combined with a Mediterranean diet intervention reduced fat mass and improved muscle mass in women affected by obesity. No improvements in grip strength were found after the interventions. However, it is crucial to emphasize that further research is needed to understand the clinical implications and assess the long-term effects of these findings.

## Figures and Tables

**Table 1:** Participants’ baseline characteristics (T_0_).

	PG (*n* = 11)	CG (*n* = 9)	*p*-Value *
Age (years)	52.0 ± 10.4	46.0 ± 6.9	0.140
Height (m)	1.62 ± 0.09	1.64 ± 0.09	0.502
Body mass (kg)	82.7 ± 13.3	94.2 ± 18.7	0.126
BMI (kg/m^2^)	31.7 ± 4.0	34.9 ± 5.2	0.138

Values are reported as means ± standard deviation (SDs).; PG, Pilates group; CG, control group; BMI, body mass index; * *p*-value from Student’s *t*-test between baseline characteristics of both groups (PG vs. CG).

**Table 2:** Participants’ body composition results assessed pre (T_0_) and post (T_1_) intervention.

	T_0_	T_1_
Body mass (kg)		
PG	82.7 ± 13.3	79.5 ± 12.6
CG	94.2 ± 18.7	90.2 ± 15.8
BMI (kg/m^2^)		
PG	31.7 ± 4.0	30.5 ± 3.9
CG	34.9 ± 5.2	33.3 ± 4.2
pFM (%)		
PG	43.6 ± 7.3	39.2 ± 7.9 *
CG	42.7 ± 6.9	41.0 ± 6.9
FFM (kg)		
PG	46.3 ± 7.5	47.6 ± 7.6 *
CG	53.1 ± 10.8	52.7 ± 10.5
ASMM (kg)		
PG	18.8 ± 3.3	19.4 ± 3.5 *
CG	21.5 ± 4.7	21.2 ± 4.7
SMM (kg)		
PG	23.9 ± 5.7	24.9 ± 5.4 *
CG	26.9 ± 7.0	26.5 ± 7.2
HGS (kg)		
PG	28.9 ± 7.3	29.0 ± 7.3
CG	34.9 ± 12.9	34.9 ± 11.4

Values are reported as means ± standard deviations (SDs); PG, Pilates group; CG, control group; BMI, Body Mass Index; pFM, percentage of fat mass; FFM, fat-free mass; ASMM, appendicular skeletal muscle mass; SMM, skeletal muscle mass; HGS, handgrip; * *p* < 0.05 vs. T_0_.

## Data Availability

The raw data supporting the conclusions of this article will be made available by the authors on request.
